# Circulating tumor DNA-guided response evaluation in patients with previously treated gastroesophageal adenocarcinoma

**DOI:** 10.1007/s10120-026-01743-w

**Published:** 2026-05-23

**Authors:** Cecilie Riis Iden, Nadia Øgaard, Salah Mohammad Mustafa, Ida Coordt Elle, Mette Yilmaz, Marianne Nordsmark, Tenna Henriksen, Torben Kruse, Mads Thomassen, Per Pfeiffer, Claus Lindbjerg Andersen, Lene Bæksgaard, Camilla Qvortrup, Morten Mau-Sørensen

**Affiliations:** 1 Department of Oncology, Copenhagen University Hospital, Rigshospitalet, Blegdamsvej 9, 2100 København Ø, Denmark; 2 Department of Molecular Medicine, Aarhus University Hospital, Aarhus,Palle Juul-Jensens Boulevard 99, 8200 Aarhus N, Denmark; 3 Department of Clinical Medicine, Faculty of Health, Aarhus University, Palle Juul-Jensens Boulevard 11, 8200 Aarhus N, Denmark; 4 Department of Clinical Genetics, Odense University Hospital, J. B. Winsløws Vej 4, 5000 Odense C, Denmark; 5 Department of Oncology, University Hospital of Aalborg, Hobrovej 18-22, 9000 Aalborg, Denmark; 6 Department of Oncology, Aarhus University Hospital, Palle Juul-Jensens Boulevard 99, 8200 Aarhus, Denmark; 7 Clinical Genome Center, Department of Clinical Research, University of Southern Denmark, Odense, Denmark; 8 Department of Oncology, Odense University Hospital, J. B. Winsløws Vej 4, 5000 Odense C, Denmark

**Keywords:** Stomach neoplasms, Esophageal neoplasms, Circulating tumor DNA, Biomarkers, Tumor, Prognosis

## Abstract

**Background:**

Systemic treatment offers only modest survival benefits in previously treated metastatic gastroesophageal adenocarcinoma (GEA). Treatment monitoring using imaging-based response assessments are often delayed and imprecise, highlighting the need for biomarkers to guide early treatment decisions. We evaluated the value of circulating tumor DNA (ctDNA) for prognosis, patient selection, and on-treatment monitoring in patients with previously treated GEA.

**Methods:**

This retrospective study aimed to investigate whether baseline ctDNA levels and early ctDNA changes assessed by ctDNA-RECIST were associated with survival outcomes in a randomized Phase 2 trial comparing trifluridine-tipiracil plus bevacizumab or trifluridine-tipiracil alone. Plasma samples were collected at baseline and during treatment (weeks 4 and 8). ctDNA was quantified by a tumor-agnostic methylation-specific digital droplet PCR (ddPCR) assay.

**Results:**

Plasma samples were available in 86 patients out of a total of 103 patients included in the trial. Patients with the highest baseline ctDNA levels (top 20%, n = 18) had significantly shorter progression-free survival (PFS; median 1.8 vs. 4.6 months; HR 2.2, *p* = 0.005) and overall survival (OS; median 5.5 vs. 9.9 months; HR 2.2, *p* = 0.004) compared to those with lower levels. Early progression by ctDNA at four weeks (HR 2.4, *p* = 0.003) and at eight weeks by radiological assessment (HR 2.9, *p* = 0.001) were both associated with inferior OS.

**Conclusions:**

ctDNA is a strong prognostic biomarker in refractory GEA. High baseline levels and early increases identify patients unlikely to benefit from treatment. Prospective validation is needed, but ctDNA monitoring may enable earlier therapeutic decision-making in clinical practice.

**Supplementary Information:**

The online version contains supplementary material available at 10.1007/s10120-026-01743-w.

## Introduction

Gastroesophageal adenocarcinoma (GEA) is a major global health concern, with more than one million new cases and nearly 800,000 deaths annually [1]. Despite major advancements in first-line therapy, including the approval of immune check-point inhibitors [2, 3], the prognosis for patients with metastatic GEA remains poor [4, 5]. Second- and third-line therapy, including irinotecan, taxanes, trifluridine/tipiracil (FTD/TPI), and ramucirumab, offer only modest survival benefits and a substantial part of patients will experience early progression [6–8]. Therefore, early identification of patients unlikely to benefit from treatment is paramount to avoid futile treatment.

Standard radiological assessment, based on the Response Evaluation Criteria in Solid Tumors (RECIST) version 1.1. criteria [9], has limited ability to detect early disease progression, as imaging is commonly performed after at least eight weeks of therapy to allow reliable assessment of tumor shrinkage. Moreover, implementing earlier imaging would place substantial additional demands on radiology resources. As a result, patients may remain on ineffective therapies longer than necessary, potentially compromising both quality of life and clinical outcomes. Therefore, identifying better tools to refine treatment decisions and improve early monitoring represents an urgent medical need.

Circulating tumor DNA (ctDNA) is a promising biomarker for real-time non-invasive disease monitoring of tumor-burden and for determining prognosis across several malignancies, including gastrointestinal cancers [10, 11]. We have previously validated a tumor-agnostic methylation assay for sensitive detection of ctDNA in patients with GEA [12]. In a separate line of work, we and others have demonstrated a strong prognostic value of ctDNA in patients with localized GEA [13–17]. However, the role of ctDNA in guiding treatment decisions based on on-treatment changes in previously treated GEA patients remains underexplored. Understanding how ctDNA dynamics correlate with treatment response could enable identification of early progressors and support timely treatment modifications. To this end, ctDNA-RECIST [18, 19] has been proposed as an exploratory framework for evaluating treatment effects based on changes in ctDNA rather than tumor size on imaging, using response classifications analogous to those of RECIST 1.1.

This study evaluated ctDNA as a biomarker in patients with previously treated, recurrent GEA, enrolled in a previously reported randomized clinical trial comparing Trifluridine/Tipiracil (FTD/TPI) alone with FTD/TPI combined with bevacizumab as second- or third-line treatment [20]. We aimed to investigate whether baseline ctDNA levels and/or early ctDNA changes assessed by ctDNA-RECIST were associated with prognosis and treatment outcomes.

## Materials and methods

### Study design

This retrospective biomarker study was conducted in patients enrolled in an open-labeled randomized, investigator-initiated trial [20]. The study was approved by the Ethics Committee of the Capital Region (H-19001247). All patients provided written informed consent.

### Patient population

Between 2019 and 2021 patients were enrolled in the Lon-Gas trial [20] at the four oncology centers treating gastroesophageal cancer in Denmark (Copenhagen University Hospital—Rigshospitalet, Aarhus University Hospital, Odense University Hospital and Aalborg University Hospital). The main study inclusion criteria were histologically confirmed GEA, ECOG performance status of 0–1, adequate organ functions, and prior treatment with fluoropyrimidine- and platinum combination chemotherapy. Exclusion criteria included known CNS metastases. Patients were randomly assigned (1:1) to receive either FTD/TPI monotherapy or FTD/TPI plus bevacizumab. Treatment cycles were repeated every four weeks. Detailed in- and exclusion criteria and treatment schedule have been published previously [20]. Patients with available plasma samples for ctDNA analysis were included in the present study.

### Plasma sample collection

Blood samples were collected at baseline, after the first and second treatment cycles (at four and eight weeks, respectively), and subsequently after every second treatment cycle (every eight weeks). Whole blood was collected in *Streck Cell-Free DNA BCT tubes* and processed within 36 h. Plasma was separated by centrifugation at 2250G for 10 min, followed by a second centrifugation at 16,000G for 10 min. The plasma supernatant was transferred to 15 mL *Corning CentriStar tubes* and stored at − 80 °C until use. Before cfDNA extraction, samples were thawed at room temperature and centrifuged at 3000G for 10 min. cfDNA was extracted immediately using the *QIAamp Circulating Nucleic Acid* Kit (Qiagen), following the manufacturer’s protocol, from a median plasma volume of 4 mL (interquartile range (IQR): 3.9–4.0 mL). The cfDNA was eluted in 60 μL and stored at − 20 °C until use (within 1–4 weeks). Quality control, after cfDNA extraction, included assessment of cfDNA concentration, cfDNA purification efficiency, and lymphocyte DNA contamination using droplet digital PCR (ddPCR). Samples with leukocyte DNA contamination were flagged, and samples with drop-outs of < 10,000 droplets on ddPCR were excluded.

### ctDNA analysis

The discovery of the methylation markers and the development and validation of the ddPCR assays used to assess ctDNA in the present study, have been described in previous studies [12, 17, 21, 22]. Also, details on cfDNA processing, including bisulfite conversion, and ddPCR analysis have been described previously [21, 22]. Briefly, cfDNA was bisulfite converted using the EZ-96 DNA Methylation-Direct MagPrep kit (Zymo Research), and bisulfite converted cfDNA was analyzed using ddPCR (BioRad) with methylation-specific assays targeting the hypermethylated promotors of *C9orf50, *CLIP4 and *KCNQ5*, and a cytosine-free (CF) control assay.

Amplitude intensity data for all individual droplets in each well in the ddPCR readout was extracted using QxManager Software (v.2.1, BioRad). The positive droplets for the three markers were summed and ctDNA concentrations including 95% confidence intervals (CIs) were calculated using Poisson statistics and adjusted for cfDNA input, cfDNA purification efficiency, bisulfite conversion recovery, and mL plasma used for analysis. ctDNA levels are reported with the unit ‘copies/mL’.

Baseline ctDNA levels were categorized as ‘high’ if they were above the 80th percentile threshold of all baseline samples (i.e. the top 20% of the patients). This cutoff was selected based on exploratory analyses of multiple thresholds [Online resource 1], with the 80th percentile providing the most distinct separation in clinical outcomes between groups.

Changes in ctDNA levels during treatment were classified according to ctDNA-RECIST criteria [18, 19]. Samples were categorized as: progressive disease (PD), if the 95% CI of the measurement was above and non-overlapping with the 95% CI of the previous measurement; stable disease (SD) if the 95% CI of the measurement overlapped with the 95% CI of the previous measurement; partial response (PR) if the 95% CI of the measurement was lower than and non-overlapping with the 95% CI of the previous measurement; near-complete response (NCR), if the 95% CI of the measurement was lower than the 95% CI of the previous measurement, and over-lapping 0; and complete response (CR), if ctDNA levels were undetectable following a previous measurement with 95% CI non-overlapping with 0. Changes in ctDNA after two treatment cycles (eight weeks) were categorized by direct comparison with baseline measurements, analogous to radiological RECIST evaluations. Overall response (OR) is defined as either CR, PR or NCR. ‘Poor ctDNA responders’ were defined as patients classified with either a ‘high’ baseline ctDNA level and/or a ctDNA increase (PD) after one treatment cycle.

All ddPCR ctDNA analyses were performed retrospectively, and classification of ctDNA changes according to ctDNA-RECIST were conducted blinded to clinical outcomes.

### Radiological response evaluation

Treatment response was assessed using Computed Tomography (CT) scans, performed at baseline and after every two cycles of therapy corresponding to every eight weeks, and evaluated per the RECIST criteria v 1.1 [9].

### Statistical analysis

OS was defined as the time from study inclusion to death of any cause. PFS was defined as the time from study inclusion to the first registration of radiological or clinical progression, or death. Patients were monitored for disease progression and survival until data cutoff (March 1st, 2023). Patients without an event were censored at the time of data cutoff. Descriptive statistics were used to summarize patient characteristics. Cox proportional hazard regression was used to assess associations between baseline ctDNA levels as a continuous variable and PFS and OS. Further, associations between dichotomized baseline ctDNA levels and clinical prognostic variables with PFS and OS were assessed using Cox proportional hazards regressions. Variables with *p* < 0.05 in the univariable analysis were retained in the multivariable models. Survival analyses were illustrated using the Kaplan–Meier method. Group differences were assessed by Cox proportional hazard regression. OS in responders compared to non-responders was assessed by a landmark analysis to account for immortal time bias, with the landmark set at 10.7 weeks, corresponding to the time point at which all response assessments by CT scans and ctDNA were completed. For all Cox proportional hazards models, the proportional hazards assumption was tested, and if violated, results from the log-rank test were reported instead. Heatmaps were created to illustrate differences between ctDNA-RECIST evaluation at four weeks and RECIST 1.1 evaluation at eight weeks, and between ctDNA-RECIST and RECIST 1.1 evaluations both performed at eight weeks.

All *p*-values were two-sided, with significance set at *p* < 0.05. Statistical analyses were performed using R version 4.3.

## Results

### Patient characteristics

**Fig. 1:**
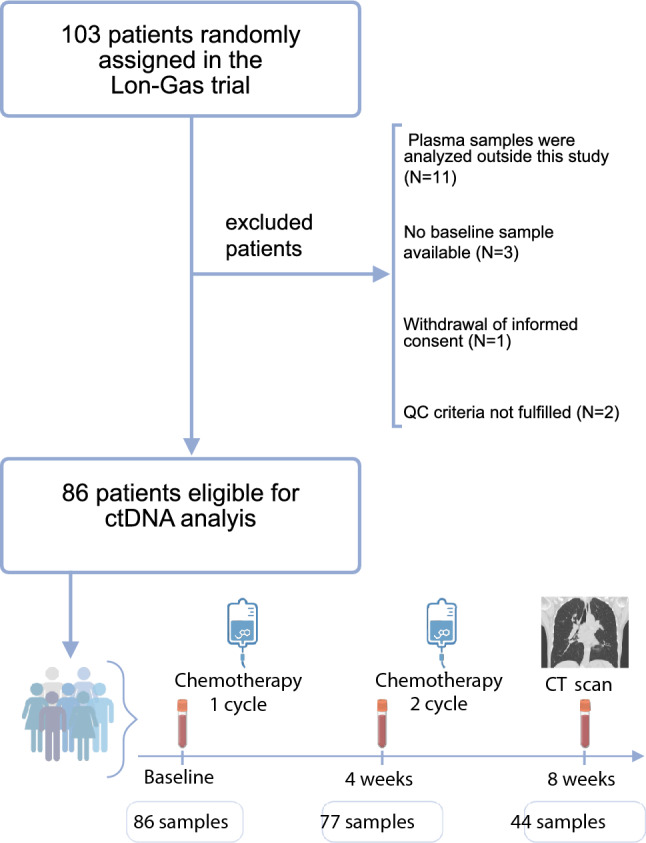
Flow chart of patient inclusion and plasma samples. Figure created using BioRender.com

A total of 103 patients were included in the Lon-Gas trial between October 1, 2019, and September 30, 2021. Of these, plasma samples for ctDNA analysis were available from 86 patients who were included in the present study (Fig. 1). The median patient age at study inclusion was 65 years, and 79% were male. Baseline patient characteristics of the ctDNA analysis cohort are summarized in Table 1 and were comparable to those of the overall Lon-Gas trial population. At the date of data cutoff (March 1, 2023), 79 patients (91%) had disease progression, and 84 (98%) had died. The median time to progression was 4.6 months (IQR: 1.9–6.6), and the median OS was 8.0 months (IQR: 4.9–12.1). Two of the 86 patients were still alive, with follow-up durations of 33 and 22 months, respectively. Median follow up time was 8.7 months (IQR: 5.1–12.9). No patients were lost to follow-up.

**Table 1:** Baseline patient characteristics of all patients enrolled in the Lon-gas trial (n = 103) and the subset with available plasma for ctDNA analysis (n = 86)

	ctDNA analysis cohort (n = 86)	Overall Lon-gas trial population (n = 103)
Age, median (IQR)	65 (42–80)	65 (56–71)
Male sex (%)	68 (79)	82 (80)
*Performance status*		
0	46 (53)	47 (46)
1	40 (47)	56 (54)
*Primary tumor location*		
Esophagus	32 (37)	41 (40)
Gastroesophageal junction	35 (41)	41 (40)
Stomach	19 (22)	21 (20)
*HER2 status*		
Negative	57 (66)	69 (67)
Positive	29 (34)	33 (32)
Inconclusive	–	1 (1)
*Number of metastatic sites*		
1	15 (18)	20 (19)
2	39 (45)	44 (43)
3 or more	32 (37)	39 (38)
*Primary tumor resection*		
Yes	22 (26)	35 (34)
No	64 (74)	68 (66)
*Treatment line*		
Second-line therapy	45 (52)	56 (54)
Third-line therapy or later	41 (48)	47 (46)
*Randomization*		
FTD/TPI	45 (52)	53 (51)
FTD/TPI + Bevacizumab	41 (48)	50 (49)
*ctDNA level at baseline*		
< 1,068 copies/mL)	68 (79)	–
> 1,068 copies/mL	18 (21)	–

#### Prognostic value of baseline ctDNA levels

**Fig. 2:**
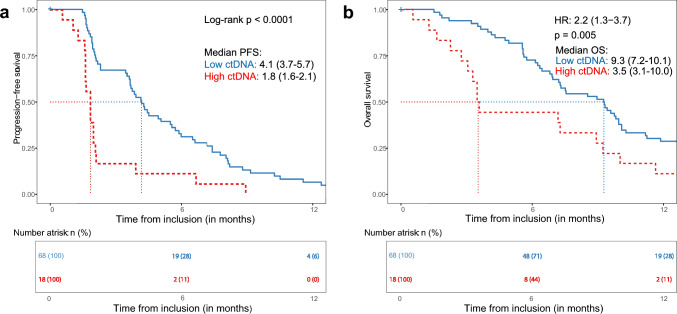
KM-plots illustrating (**a**) PFS and **b** OS for patients with high ctDNA levels (> 80th percentile threshold of 1,068 copies/mL, n = 18, red line) versus low ctDNA levels (< 80th percentile threshold, < 1068 copies/mL n = 68, blue line) at baseline

To assess the prognostic value of baseline ctDNA, we performed a Cox proportional hazard analysis with log-transformed baseline ctDNA levels as a continuous variable. Higher baseline ctDNA levels were significantly associated with shorter PFS (HR = 1.1; 95% CI, 1.0–1.2; *p* = 0.016), whereas no significant association was observed with OS. To further explore the associations between baseline ctDNA levels and outcome, we performed exploratory analyses of multiple cutoffs to separate baseline ctDNA levels into distinct groups to correlate to outcome [Online resource 1]. The median baseline level of ctDNA was 64.4 copies/mL. Patients in the lowest decile (< 1.2 copies/mL, the 10th percentile) had both longer PFS and OS (non-significant) compared to the patients with higher ctDNA levels [Online resource 2]. Conversely, the 10% of patients with the highest baseline ctDNA levels (i.e. > 2029 copies/mL, the 90th percentile) experienced shorter PFS and OS compared to those with lower levels. For further analyses, the 80th percentile cutoff (corresponding to 1068 copies/mL) was selected to define ‘high’ versus ‘low’ baseline ctDNA groups. Patients with high baseline ctDNA levels (> 1,068 copies/mL, n = 18) had significantly shorter PFS (median 1.8 vs. 4.2 months; log-rank *p* < 0.001; (Fig. 2a)) and OS (median 3.5 vs. 9.3 months; *p* = 0.005, (Fig. 2b)) than the patients with lower levels. Univariable Cox regression analysis (Table 2) demonstrated that high baseline ctDNA was significantly associated with worse OS (*p* = 0.005) and PFS (*p* < 0.001). In multivariable Cox regression analysis, high baseline ctDNA remained independently associated with worse OS (HR 2.2; 95% CI, 1.2–3.8; *p* = 0.0039) and PFS (HR 2.9; 95% CI, 1.9–4.5; *p* < 0.001).

**Table 2:** Univariable and multivariable Cox regression analyses.

	Univariable analysis				Multivariable analysis	
Variable	OS, n = 86		PFS, n = 86		OS, n = 86	
	HR (95% CI)	*p*-value	HR (95% CI)	*p*-value	HR (95% CI)	*p*-value
Age (continuous)	1.0 (0.9–1.1)	0.94	1.0 (0.9–1.1)	0.19		
*ctDNA level at baseline*						
Low	Reference		Reference		Reference	
High	2.2 (1.3–3.7)	**0.005**	2.9 (1.9–4.5)	** < 0.001**	2.2 (1.2–3.8)	**0.0039**
*Performance status*						
0	Reference		Reference			
1	1.3 (0.8–2.0)	0.27	1.3 (0.8–2.0)	0.27		
*HER-2 status*						
Negative	Reference		Reference			
Positive	0.5 (0.5–1.4)	0.50	1.1 (0.7–1.7)	0.79		
*Primary tumor resection*						
No resection	Reference		Reference			
Resection	0.8 (0.5–1.3)	0.32	0.9 (0.5–1.5)	0.59		
*Line of treatment*						
First or second	Reference		Reference			
Third line or later	1.3 (0.8–2.0)	0.23	0.8–1.9	0.36		
*Liver metastases*						
No	Reference		Reference			
Yes	1.4 (0.9–2.2)	0.12	1.3 (0.8–2.0)	0.32		
*Number of metastatic sites*						
1	Reference		Reference		Reference	
> 1	1.3 (1.0–1.7)	**0.014**	1.1 (0.9–1.3)	0.57	1.3 (1.1–1.7)	**0.012**
*Randomization*						
FTD/TPI	Reference		Reference			
FTD/TPI + Bevacizumab	0.8 (0.5–1.3)	0.84	0.8 (0.5–1.2)	0.25		

A ‘High’ baseline ctDNA level was defined as > 80th percentile (1,068 copies/mL); patients with ctDNA levels below this threshold were classified as ‘Low’ baseline ctDNA. Variables with *p* < 0.05 in the univariable analyses were entered into the multivariable models. *P*-values below this threshold are marked in bold

Bold hightlights significant p-values below 0.05

### ctDNA dynamics by use of ctDNA-RECIST

Among the 77 patients with plasma available after the first treatment cycle (4 weeks) PD, SD, and OR (PR, NCR or CR) were recorded in 18 (23%), 25 (33%), and 34 (44%) patients, respectively, according to the ctDNA-RECIST criteria [18, 19]. Of the 44 patients with available plasma after the second treatment cycle (8 weeks), 8 (18%), 17 (39%), and 19 (43%) had PD, SD, and OR, respectively.

**Fig. 3:**
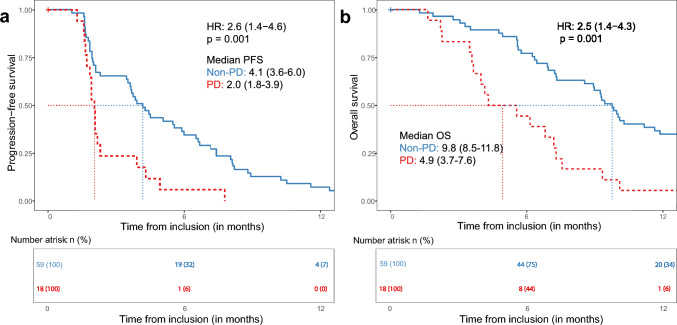
KM-plots illustrating (**a**) PFS and **b** OS for patients with ctDNA PD (n = 18, red line) versus non-PD (n = 59, blue line) from baseline to after one treatment cycle (4 weeks)

To study the association of ctDNA dynamics with clinical outcome, patients were grouped into ‘ctDNA PD’ and ‘ctDNA non-PD’ categories for survival analysis. The ctDNA non-PD group included patients with the ctDNA-RECIST categories: SD, PR, NCR, and CR. After one treatment cycle (4 weeks), patients with ctDNA PD had a significantly shorter PFS (2.0 months), compared to the ctDNA non-PD group (4.1 months) (HR: 2.6; 95% CI: 1.4–4.6; *p* = 0.001) (Fig. 3a). Also, OS was significantly shorter for the ctDNA PD group (4.9 months) than for the ctDNA non-PD group (9.8 months) (HR: 2.5; 95% CI: 1.4–4.3; *p* = 0.001) (Fig. 3b). Although only 44 patients were evaluable after two treatment cycles (8 weeks), ctDNA-RECIST response evaluation at this timepoint, supported the findings, showing that patients with ctDNA PD had an inferior PFS (HR: 2.2; 95% CI: 0.9–5.4, *p* = 0.06) and OS (HR: 1.3; 95% CI: 0.6–2.8; *p* = 0.5) compared to the ctDNA non-PD group [Online resource 3a–b].

To explore whether ctDNA could identify a subgroup of patients with particularly poor outcomes, patients were classified as poor or non-poor ctDNA responders [Online Resource 4a–b]. Poor ctDNA responders had significantly shorter PFS and OS compared with non-poor responders (median PFS, 1.9 vs 4.8 months; median OS, 5.6 vs 9.9 months; both *P* < 0.001).

#### Comparison of ctDNA-RECIST to RECIST v 1.1

**Fig. 4:**
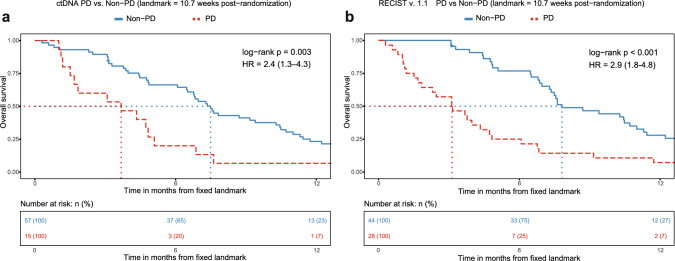
KM-plots illustrating OS according to disease status assessed by (**a**) RECIST v1.1 after two treatment cycles and by (**b**) ctDNA-RECIST after one treatment cycle. The landmark was set at 10.7 weeks, corresponding to the time of the latest radiological evaluation, to account for potential immortal time bias

Thirty-two (41%), 42 (56%), and 2 (3%) patients achieved PD, SD, and OR at 8 weeks according to standard-of-care radiological evaluation (RECIST v 1.1). To assess how early ctDNA-RECIST response (4 weeks and 8 weeks, respectively) compared to RECIST v 1.1. at 8 weeks, we aligned the ctDNA-RECIST and the RECIST v 1.1 response evaluations for each patient. PD at 8 weeks by radiological assessment seem to identify patients with very short survival more often that PD at 4 weeks by ctDNA [Online resource 5a]. The overall percent agreement was modest (68%) between PD assessed by ctDNA at 4 weeks compared to CT scans at 8 weeks. Agreement was fair according to Cohen’s kappa coefficient of 0.29 [Online resource 6]. However, landmark analyses demonstrated that patients classified as PD by either RECIST v 1.1 or ctDNA-RECIST had significantly shorter OS than those without PD. HRs for the two methods were in similar range (2.9 versus 2.4) and with overlapping confidence interval even though ctDNA response assessment occurred 4 weeks earlier (Fig. 4a–b).

## Discussion

This retrospective study of 86 patients with previously treated GEA demonstrates that both high baseline ctDNA levels and early increases in ctDNA after treatment initiation are associated with significantly worse outcomes, potentially identifying patients who are unlikely to benefit from treatment. These findings build upon prior work establishing the prognostic value of ctDNA in gastrointestinal cancers.

GEA remains a challenging disease with limited effective therapies beyond first line. Timely identification of patients unlikely to benefit from ongoing treatment is critical as a substantial proportion of patients experience early progression. In fact, up to 40% of patients included in the Lon-Gas trial went off-study due to progression after only eight weeks of treatment in line with what has been reported in other studies [23, 24]. Identifying early progressors will enable switch of therapy (if possible) or to offer the patient palliative care to avoid burdensome adverse effects of futile treatment and enhance the patient’s quality of life during the last phase of life.

Conventional treatment monitoring by imaging has several limitations. First, far from all patients present with measurable disease as defined by RECIST v1.1 making them ineligible for clinical trials according to commonly used inclusion criteria. Also, a certain time on treatment is needed to accurately assess changes in tumor size. In addition, response rates are low in the range of 0–11% [23, 24] although addition of ramucirumab to chemotherapy has resulted in response rates of 25% [25]. Patients who achieve stable disease present a large diverse population, including both short- and long-term survivors. Furthermore, the equipment and personnel resources required for imaging limit how frequently scans can be performed. In contrast, ctDNA-based assessment offers a real-time, patient-convenient and more accessible method for response evaluation. Our data suggest that ctDNA-based evaluation may provide a more dynamic prediction of response and outcome, which may complement conventional imaging-based evaluation by capturing molecular changes earlier in the treatment course. ctDNA progression (measured at four weeks) stratified OS to a similar extent as radiological progression assessed several weeks later, indicating that ctDNA can provide clinically relevant prognostic information well before the first imaging evaluation (Fig. 4a–b). Further, in this study, changes in ctDNA levels after just one treatment cycle were predictive of outcomes, underscoring ctDNA’s potential role in early decision-making. The non-invasive nature of ctDNA monitoring, together with its suitability for repeated assessments, enables continuous evaluation of tumor burden and treatment response. These findings suggest that integrating ctDNA into routine practice could guide treatment discontinuation, avoid unnecessary toxicity, and support earlier transition to alternative strategies in molecular non-responders.

Supporting the findings of our study, other studies have investigated absolute baseline ctDNA levels in previously treated gastrointestinal cancers and their correlations to clinical outcome. Vandeputte et al. [26] demonstrated that baseline cfDNA concentrations above the median cutoff were associated with shorter OS in patients with metastatic colorectal cancer. In smaller cohorts of metastatic GEA patients, both Openshaw et al. [27] and Tougeron et al. [28] have shown that patients with high baseline ctDNA levels (cutoffs of 61 copies/mL and 333 copies/mL, respectively) had a reduced OS.

Our findings contribute to the growing body of evidence suggesting that early molecular response, as measured by ctDNA, may serve as an early predictor of treatment response potentially supplementing or even bypassing conventional radiological imaging. In a small study presented at ASCO GI 2025, Chakrabarti et al. [29] demonstrated that gastrointestinal cancer patients whose ctDNA levels increased after one cycle of immunotherapy were uniformly found to have progressive disease on imaging, identifying them as poor ctDNA-responders. Similarly, a prospective study by Tatalovic et al. [30] showed that an early drop in ctDNA levels, within two weeks of initiating treatment, was associated with improved outcomes in patients with metastatic GEA, while the absence of such a decline correlated with poor prognosis. Recently, Tougeron et al. reported that after the second cycle of treatment for patients with advanced GEA, a ctDNA decrease of less than 75% compared to the baseline level was correlated to shorter PFS and OS compared to the patients with > 75% decrease [28]. Although conducted in smaller cohorts and using less standardized approaches to determine ctDNA dynamics than ctDNA-RECIST, these studies consistently support our findings, reinforcing the potential prognostic value of early ctDNA changes during treatment.

Now, larger prospective trials incorporating ctDNA-guided treatment adaptation are needed to establish clinical utility. Defining universal response thresholds and integrating ctDNA with radiologic and clinical parameters may ultimately enable a more personalized, responsive approach to treatment in advanced GEA.

In the present study, several limitations merit consideration. Firstly, the relatively small sample size and limited availability of on-treatment samples may have impacted the robustness of our findings, especially for the analyses of the limited number of samples after second cycle of treatment. Thus, the heavy reduction of samples during treatment do reflect a real challenge in this clinical setting, as many patients progress or get too ill before they complete the second cycle of treatment. Secondly, the definition of high baseline ctDNA based on the 80th percentile cutoff, while data-driven, is cohort-specific, and hence, may not be broadly applicable and requires external validation in independent cohorts.

## Conclusions

ctDNA is a robust prognostic biomarker in patients with previously treated GEA. High baseline levels and early on-treatment increases were independently associated with poor outcomes and identified patients unlikely to benefit from continued therapy. These findings support the integration of ctDNA monitoring into clinical practice as a tool for early response assessment and treatment adaptation. Prospective validation in larger cohorts is warranted to define standardized thresholds and confirm the clinical utility of ctDNA-guided strategies.

## Supplementary Information

Below is the link to the electronic supplementary material.

Supplementary file 1 (PDF 543 kb)
ctDNA levels of the 86 baseline samples and percentile thresholds. The 80th percentile threshold (red line) was selected to define ’high’ versus ’low’ baseline ctDNA groups. Dashed lines indicate the median, and the 10th and 90th percentile thresholds.

Supplementary file 2 (PDF 761 kb)
Survival analyses based on different thresholds of ctDNA levels at baseline. KM-plots of (a) PFS and (b) OS for patients with baseline ctDNA below the 10th percentile (<1.2 copies/mL; n = 9, blue line) versus above (n = 77, red line), and (c) PFS and (d) OS for patients with baseline ctDNA level below the median (<64 copies/mL; n =43, blue line) versus above (n =43, red line), and (e) PFS and (f) OS for patients with baseline ctDNA above the 90th percentile (>2049 copies/mL; n = 9, red line) versus below (n = 77, blue line).

Supplementary file 3 (PDF 340 kb)
KM-plots illustrating (a) PFS and (b) OS for patients with ctDNA PD (n = 8, red line) versus non-PD (n = 36, blue line) from baseline to after two treatment cycles (eight weeks).

Supplementary file 4 (PDF 327 kb)
KM-plots illustrating (a) PFS and (b) OS for poor ctDNA responders (n = 31, red line) versus non-poor ctDNA responders (n = 48, blue line).

Supplementary file 5 (PDF 179 kb)
Heatmaps comparing (a) radiological response (RECIST v1.1) after twotreatment cycles (eight weeks) with ctDNA RECIST classifications assessed after one treatment cycle (four weeks) (patients n = 77), and (b) radiological response after two treatment cycles (RECIST v1.1) with ctDNA RECIST classifications assessed after two treatment cycles (eight weeks) (patients n = 44). Each column represents an individual patient. Survival outcomes are sorted and colored (the darker color the shorter OS).

Supplementary file 6 (PDF 33 kb)
Table showing agreement between radiological response assessed by CT according to RECIST v1.1 and ctDNA response assessed by ctDNA-RECIST. As CT imaging cannot be considered a definitive gold standard, agreement between the two methods was evaluated using overall percent agreement, positive and negative agreement, and Cohen’s kappa coefficient rather than sensitivity and specificity.

## References

[1] Sung H, Ferlay J, Siegel RL, Laversanne M, Soerjomataram I, Jemal A, et al. Global cancer statistics 2020: GLOBOCAN estimates of incidence and mortality worldwide for 36 cancers in 185 countries. CA Cancer J Clin. 2021;71(3):209–49. 33538338 10.3322/caac.21660

[2] Janjigian YY, Shitara K, Moehler M, Garrido M, Salman P, Shen L, et al. First-line nivolumab plus chemotherapy versus chemotherapy alone for advanced gastric, gastro-oesophageal junction, and oesophageal adenocarcinoma (CheckMate 649): a randomised, open-label, phase 3 trial. Lancet. 2021;398(10294):27–40. 34102137 10.1016/S0140-6736(21)00797-2 PMC8436782

[3] Sun JM, Shen L, Shah MA, Enzinger P, Adenis A, Doi T, et al. Pembrolizumab plus chemotherapy versus chemotherapy alone for first-line treatment of advanced oesophageal cancer (KEYNOTE-590): a randomised, placebo-controlled, phase 3 study. Lancet. 2021;398(10302):759–71. 34454674 10.1016/S0140-6736(21)01234-4

[4] Obermannová R, Alsina M, Cervantes A, Leong T, Lordick F, Nilsson M, et al. Oesophageal cancer: ESMO clinical practice guideline for diagnosis, treatment and follow-up. Ann Oncol. 2022;33(10):992–1004. 35914638 10.1016/j.annonc.2022.07.003

[5] Lordick F, Carneiro F, Cascinu S, Fleitas T, Haustermans K, Piessen G, et al. Gastric cancer: ESMO clinical practice guideline for diagnosis, treatment and follow-up. Ann Oncol. 2022;33(10):1005–20. 35914639 10.1016/j.annonc.2022.07.004

[6] Hsu A, Zayac AS, Eturi A, Almhanna K. Treatment for metastatic adenocarcinoma of the stomach and gastroesophageal junction: 2020. Ann Transl Med. 2020;8(17):1109–1109. 33145328 10.21037/atm-20-1159 PMC7575962

[7] ter Veer E, Haj Mohammad N, van Valkenhoef G, Ngai LL, Mali RMA, van Oijen MGH, et al. Second- and third-line systemic therapy in patients with advanced esophagogastric cancer: a systematic review of the literature. Cancer Metastasis Rev. 2016;35(3):439–56. 27417221 10.1007/s10555-016-9632-2 PMC5035657

[8] Shitara K, Doi T, Dvorkin M, Mansoor W, Arkenau HT, Prokharau A, et al. Trifluridine/tipiracil versus placebo in patients with heavily pretreated metastatic gastric cancer (TAGS): a randomised, double-blind, placebo-controlled, phase 3 trial. Lancet Oncol. 2018;19(11):1437–48. 30355453 10.1016/S1470-2045(18)30739-3

[9] Eisenhauer EA, Therasse P, Bogaerts J, Schwartz LH, Sargent D, Ford R, et al. New response evaluation criteria in solid tumours: revised RECIST guideline (version 1.1). Eur J Cancer. 2009;45(2):228–47. 19097774 10.1016/j.ejca.2008.10.026

[10] Moati E, Taly V, Garinet S, Didelot A, Taieb J, Laurent‐puig P, et al. Role of circulating tumor dna in gastrointestinal cancers: Current knowledge and perspectives. Vol. 13, Cancers. MDPI; 2021 10.3390/cancers13194743 PMC8507552 34638228

[11] Kirchweger P, Wundsam HV, Rumpold H. Circulating tumor DNA for diagnosis, prognosis and treatment of gastrointestinal malignancies. World J Clin Oncol. 2022;13(6):473–84. 35949436 10.5306/wjco.v13.i6.473 PMC9244970

[12] Øgaard N, Iden CR, Jensen SØ, Mustafa SM, Aagaard E, Bramsen JB, et al. DNA methylation markers for sensitive detection of circulating tumor DNA in patients with gastroesophageal cancers. ESMO Gastrointestinal Oncology [Internet]. 2024 Dec;6:100104. Available from: https://linkinghub.elsevier.com/retrieve/pii/S2949819824000657 10.1016/j.esmogo.2024.100104 PMC12836704 41646953

[13] Kim YW, Kim YH, Song Y, Kim HS, Sim HW, Poojan S, et al. Monitoring circulating tumor DNA by analyzing personalized cancer-specific rearrangements to detect recurrence in gastric cancer. Exp Mol Med. 2019;51(8):1–10. 31395853 10.1038/s12276-019-0292-5 PMC6802636

[14] Ococks E, Frankell AM, Masque Soler N, Grehan N, Northrop A, Coles H, et al. Longitudinal tracking of 97 esophageal adenocarcinomas using liquid biopsy sampling. Ann Oncol. 2021;32(4):522–32. 33359547 10.1016/j.annonc.2020.12.010

[15] Leal A, van Grieken NCT, Palsgrove DN, Phallen J, Medina JE, Hruban C, et al. White blood cell and cell-free DNA analyses for detection of residual disease in gastric cancer. Nat Commun. 2020;11(1):525. 31988276 10.1038/s41467-020-14310-3 PMC6985115

[16] Maron SB, Chase LM, Lomnicki S, Kochanny S, Moore KL, Joshi SS, et al. Circulating tumor DNA sequencing analysis of gastroesophageal adenocarcinoma. Clin Cancer Res. 2019;25(23):7098–112. 31427281 10.1158/1078-0432.CCR-19-1704 PMC6891164

[17] Iden CR, Mustafa SM, Øgaard N, Henriksen T, Jensen SØ, Ahlborn LB, et al. Circulating tumor DNA predicts recurrence and survival in patients with resectable gastric and gastroesophageal junction cancer. Gastric Cancer. 2025. 10.1007/s10120-024-01556-9. 39369091 10.1007/s10120-024-01556-9 PMC11706848

[18] Jakobsen AKM, Spindler KLG. ctDNA-Response evaluation criteria in solid tumors - a new measure in medical oncology. Eur J Cancer. 2023;180:180–3. 36610263 10.1016/j.ejca.2022.11.039

[19] Spindler KLG, Jakobsen A. Circulating tumor DNA: Response evaluation criteria in solid tumors–can we RECIST? Focus on colorectal cancer. Vol. 15, Therapeutic Advances in Medical Oncology. SAGE Publications Inc.; 2023 10.1177/17588359231171580 PMC10154995 37152423

[20] Baeksgaard Jensen L, Yilmaz M, Nordsmark M, Möller S, Elle IC, Ladekarl M, et al. TRIFLURIDINE/TIPIRACIL (FTD/TPI) with or without bevacizumab in previously treated patients with esophago-gastric adenocarcinoma, a randomised phase III trial. EClinicalMedicine. 2024;1:70. 10.1016/j.eclinm.2024.102521 PMC10940909 38495525

[21] Jensen SØ, Øgaard N, Ørntoft MBW, Rasmussen MH, Bramsen JB, Kristensen H, et al. Novel DNA methylation biomarkers show high sensitivity and specificity for blood-based detection of colorectal cancer—a clinical biomarker discovery and validation study. Clin Epigenetics. 2019;11(1):158. 31727158 10.1186/s13148-019-0757-3 PMC6854894

[22] Jensen SØ, Øgaard N, Nielsen HJ, Bramsen JB, Andersen CL. Enhanced performance of DNA methylation markers by simultaneous measurement of sense and antisense DNA strands after cytosine conversion. Clin Chem. 2020;66(7):925–33. 32460325 10.1093/clinchem/hvaa100

[23] Thuss-Patience PC, Kretzschmar A, Bichev D, Deist T, Hinke A, Breithaupt K, et al. Survival advantage for irinotecan versus best supportive care as second-line chemotherapy in gastric cancer–a randomised phase III study of the Arbeitsgemeinschaft Internistische Onkologie (AIO). Eur J Cancer. 2011;47(15):2306–14. 21742485 10.1016/j.ejca.2011.06.002

[24] Ford H, Gounaris I. Docetaxel and its potential in the treatment of refractory esophagogastric adenocarcinoma. Therap Adv Gastroenterol. 2015;8(4):189–205. 26136837 10.1177/1756283X15585468 PMC4480574

[25] Wilke H, Muro K, Van Cutsem E, Oh SC, Bodoky G, Shimada Y, et al. Ramucirumab plus paclitaxel versus placebo plus paclitaxel in patients with previously treated advanced gastric or gastro-oesophageal junction adenocarcinoma (RAINBOW): a double-blind, randomised phase 3 trial. Lancet Oncol. 2014;15(11):1224–35. 25240821 10.1016/S1470-2045(14)70420-6

[26] Vandeputte C, Kehagias P, El Housni H, Ameye L, Laes JF, Desmedt C, et al. Circulating tumor DNA in early response assessment and monitoring of advanced colorectal cancer treated with a multi-kinase inhibitor. Oncotarget. 2018;9(25):17756–69. 29707145 10.18632/oncotarget.24879 PMC5915153

[27] Openshaw MR, Mohamed AA, Ottolini B, Fernandez-Garcia D, Richards CJ, Page K, et al. Longitudinal monitoring of circulating tumour DNA improves prognostication and relapse detection in gastroesophageal adenocarcinoma. Br J Cancer. 2020;123(8):1271–9. 32719550 10.1038/s41416-020-1002-8 PMC7555811

[28] Tougeron D, Louvet C, Desramé J, Evesque L, Angelergues A, Carnot A, et al. Circulating tumor DNA strongly predicts efficacy of chemotherapy plus immune checkpoint inhibitors in patients with advanced gastro-esophageal adenocarcinoma. Communications Medicine. 2025;5(1):136. 40275077 10.1038/s43856-025-00867-x PMC12022060

[29] Chakrabarti S, Bajor DL, Lumish MA, Conces M, Mohamed A, Mahipal A, et al. Short interval circulating tumor DNA (ctDNA) kinetics as a predictor of tumor re-sponse in patients with gastrointestinal (GI) cancer receiving immune checkpoint inhibitor (ICI)-based treatment. 2025.

[30] Tatalovic S, Doleschal B, Kupferthaler A, Grundner S, Burghofer J, Webersinke G, et al. Circulating tumor DNA (ctDNA) dynamics predict early response to treatment in metastasized gastroesophageal cancer (mGEC) after 2 weeks of systemic treatment. Cancers. 2024;16(23):3960. 39682148 10.3390/cancers16233960 PMC11639943

