# Supplementary figures and images for: Circulating tumor DNA-guided response evaluation in patients with previously treated gastroesophageal adenocarcinoma
Source: Gastric Cancer. 2026 May 23;29(4):798–807. doi: 10.1007/s10120-026-01743-w (PMC13315376; doi:10.1007/s10120-026-01743-w)

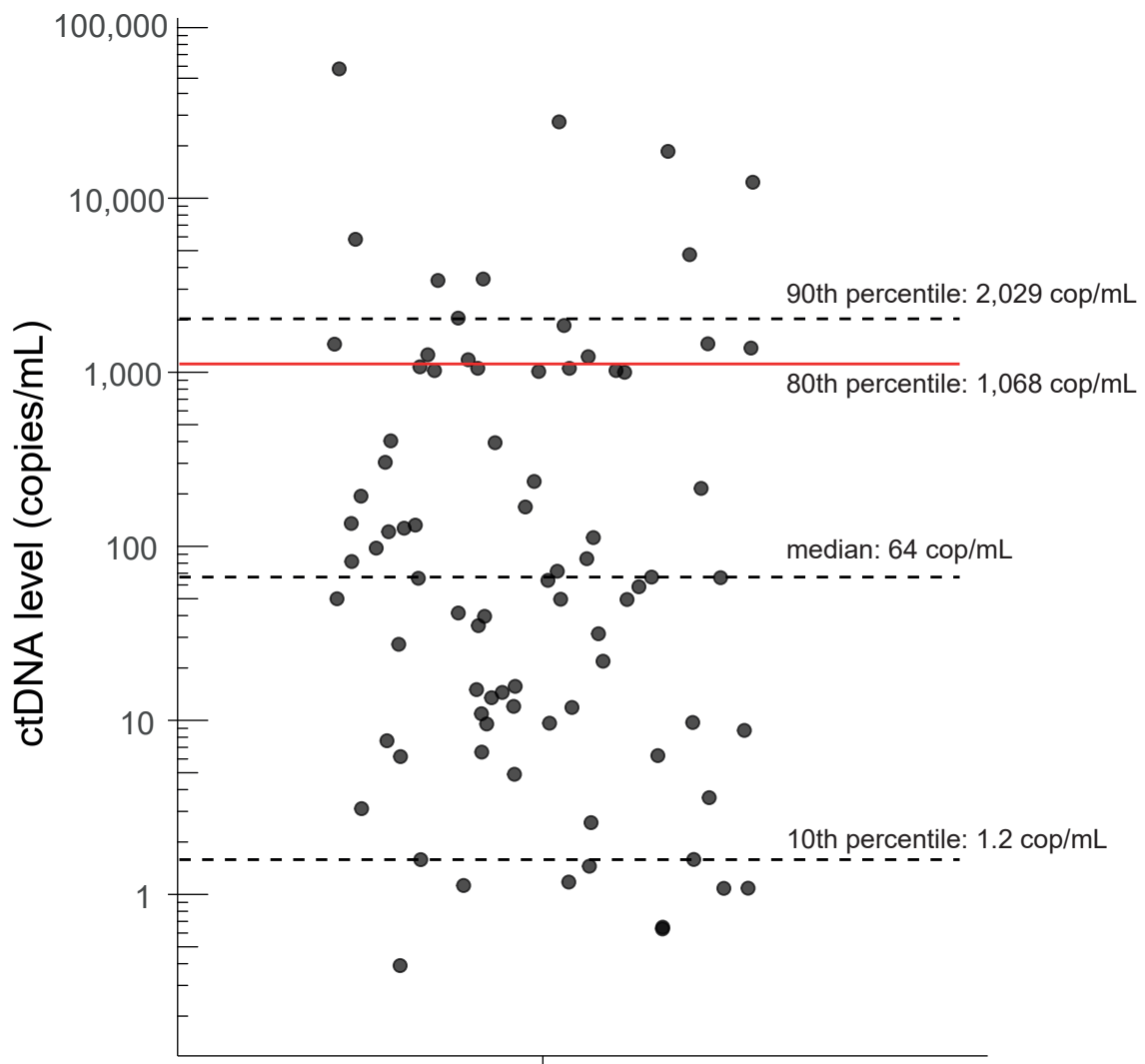

Supplement: Supplementary file 1 — (PDF 543 kb) ctDNA levels of the 86 baseline samples and percentile thresholds. The 80th percentile threshold (red line) was selected to define ’high’ versus ’low’ baseline ctDNA groups. Dashed lines indicate the median, and the 10th and 90th percentile thresholds. [file 10120_2026_1743_MOESM1_ESM.pdf]

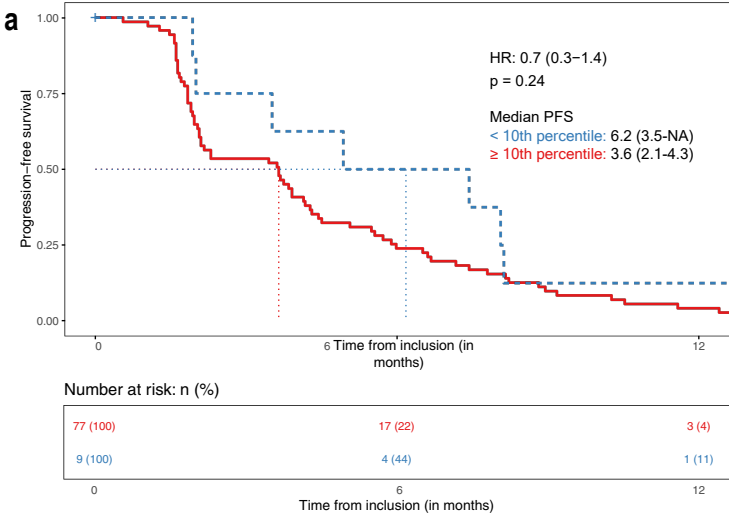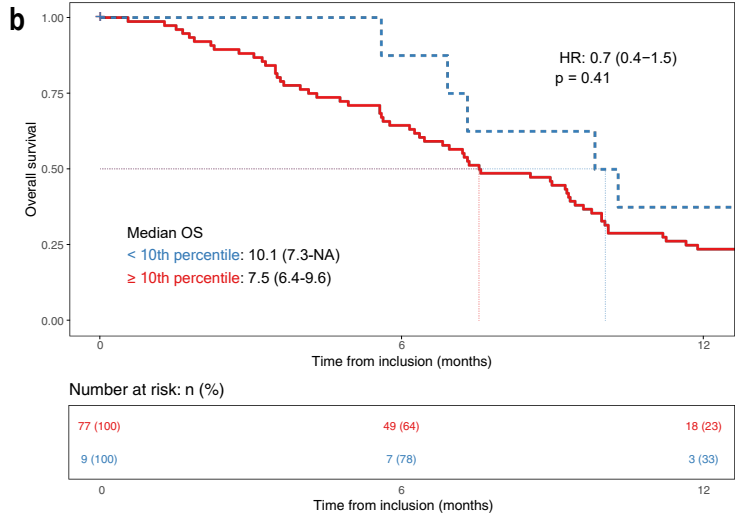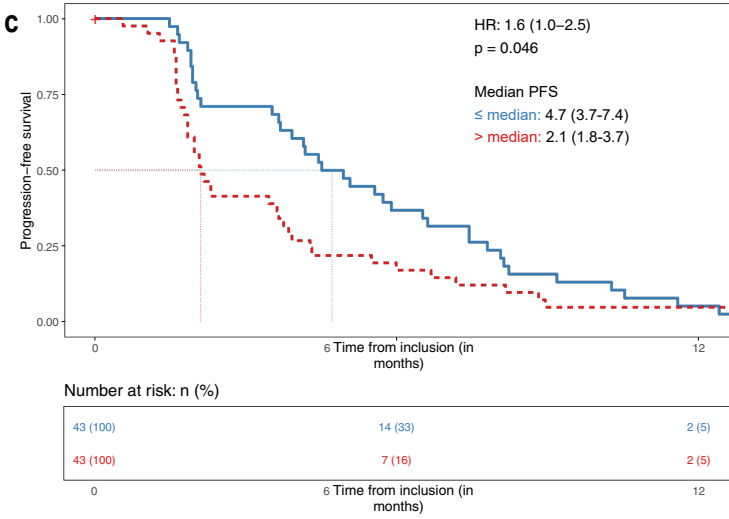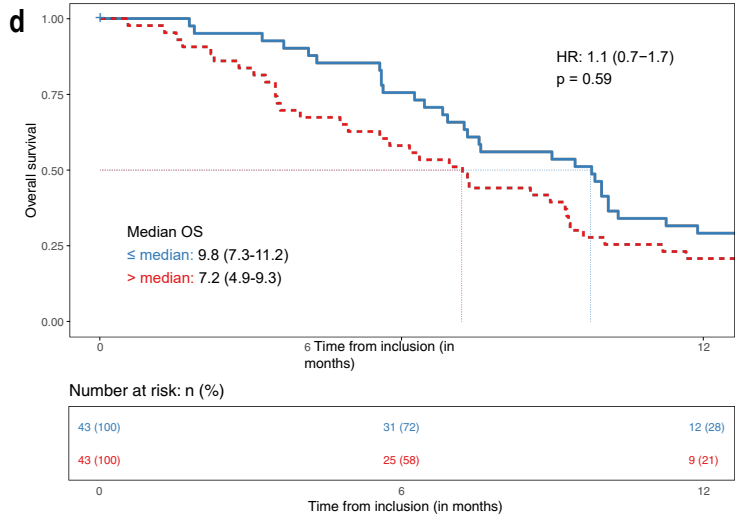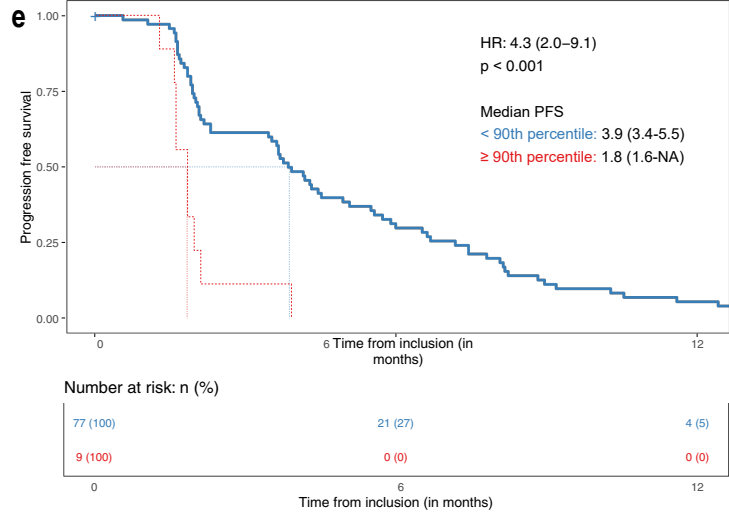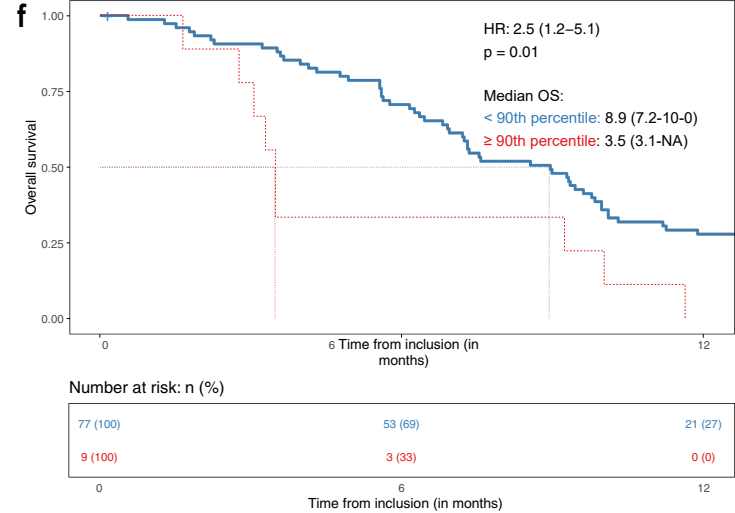

Supplement: Supplementary file 2 — (PDF 761 kb) Survival analyses based on different thresholds of ctDNA levels at baseline. KM-plots of (a) PFS and (b) OS for patients with baseline ctDNA below the 10th percentile (<1.2 copies/mL; n = 9, blue line) versus above (n = 77, red line), and (c) PFS and (d) OS for patients with baseline ctDNA level below the median (<64 copies/mL; n =43, blue line) versus above (n =43, red line), and (e) PFS and (f) OS for patients with baseline ctDNA above the 90th percentile (>2049 copies/mL; n = 9, red line) versus below (n = 77, blue line). [file 10120_2026_1743_MOESM2_ESM.pdf]

**a**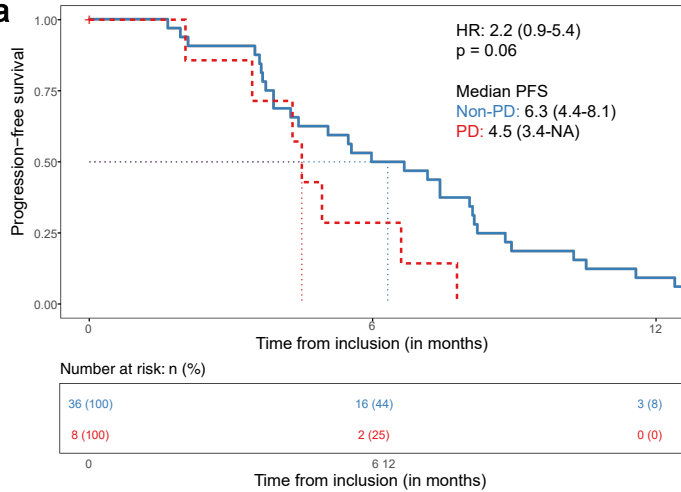**b**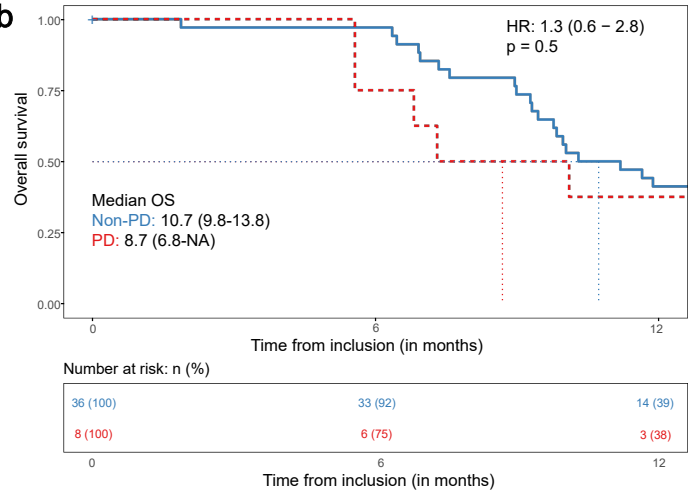

Supplement: Supplementary file 3 — (PDF 340 kb) KM-plots illustrating (a) PFS and (b) OS for patients with ctDNA PD (n = 8, red line) versus non-PD (n = 36, blue line) from baseline to after two treatment cycles (eight weeks). [file 10120_2026_1743_MOESM3_ESM.pdf]

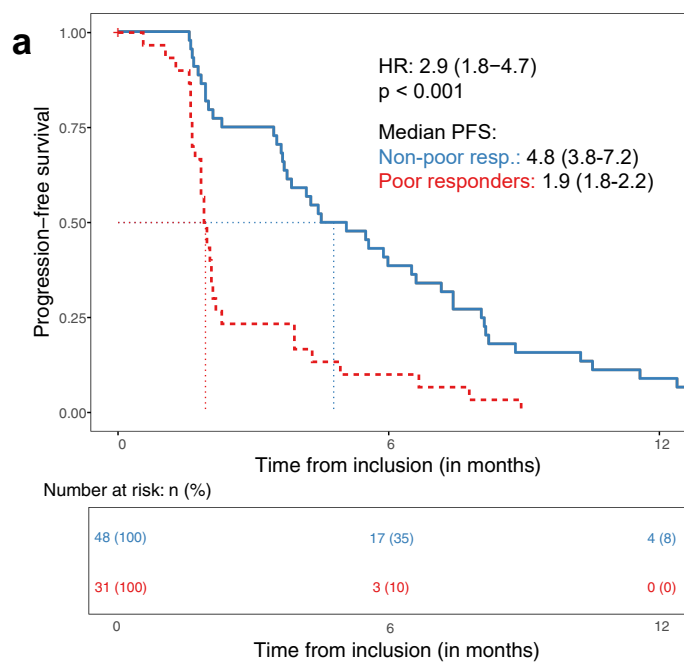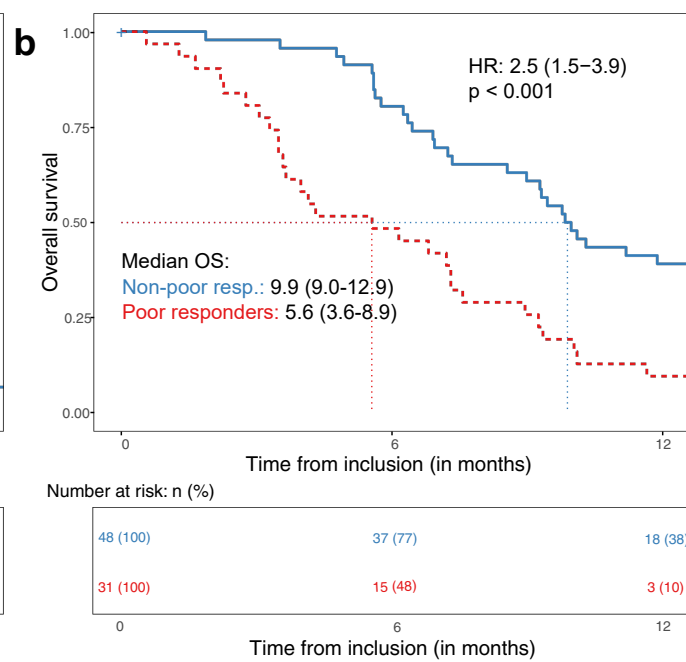

Supplement: Supplementary file 4 — (PDF 327 kb) KM-plots illustrating (a) PFS and (b) OS for poor ctDNA responders (n = 31, red line) versus non-poor ctDNA responders (n = 48, blue line). [file 10120_2026_1743_MOESM4_ESM.pdf]

**a**

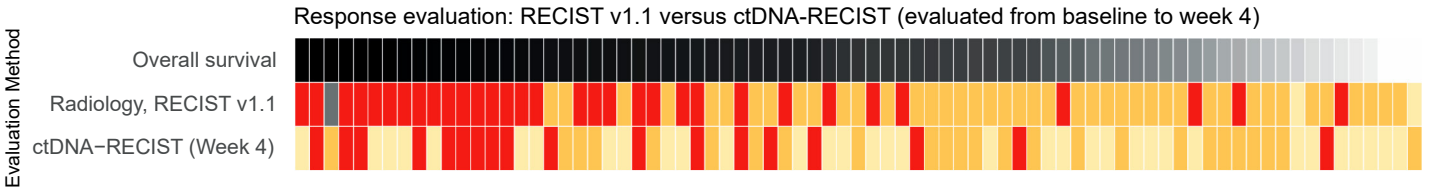

**b**

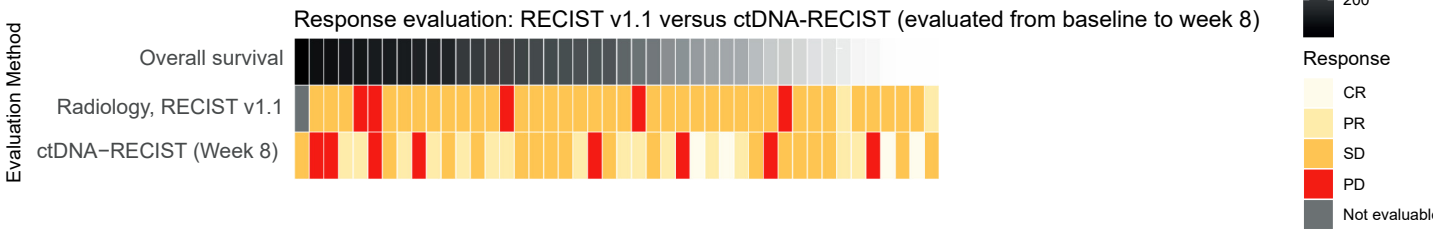

Supplement: Supplementary file 5 — (PDF 179 kb) Heatmaps comparing (a) radiological response (RECIST v1.1) after twotreatment cycles (eight weeks) with ctDNA RECIST classifications assessed after one treatment cycle (four weeks) (patients n = 77), and (b) radiological response after two treatment cycles (RECIST v1.1) with ctDNA RECIST classifications assessed after two treatment cycles (eight weeks) (patients n = 44). Each column represents an individual patient. Survival outcomes are sorted and colored (the darker color the shorter OS). [file 10120_2026_1743_MOESM5_ESM.pdf]
