# Supplementary material for: Circulating tumor DNA-guided response evaluation in patients with previously treated gastroesophageal adenocarcinoma
Source: Gastric Cancer. 2026 May 23;29(4):798–807. doi: 10.1007/s10120-026-01743-w (PMC13315376; doi:10.1007/s10120-026-01743-w)
Supplement: Supplementary file 6 — (PDF 33 kb) Table showing agreement between radiological response assessed by CT according to RECIST v1.1 and ctDNA response assessed by ctDNA-RECIST. As CT imaging cannot be considered a definitive gold standard, agreement between the two methods was evaluated using overall percent agreement, positive and negative agreement, and Cohen’s kappa coefficient rather than sensitivity and specificity. [file 10120_2026_1743_MOESM6_ESM.pdf]

| | CT PD | CT nonPD | | |
|---|---|---|---|---|
| **ctDNA PD** | 12 | 5 | 17 | |
| **ctDNA nonPD** | 19 | 38 | 57 | |
| | 31 | 43 | | 74 |
| | | | 74 | |

| | | | | |
|---|---|---|---|---|
| **Positive percent agreement (PPA)** | 0,39 | | | |
| **Negative percent agreement (NPA)** | | 0,88 | | |
| **Overall percent agreement (OPA)** | | | | 0,68 |

| | |
|---|---|
| $p_0$ | 0,68 |
| $p_{PD}$ | 0,10 |
| $p_{nonPD}$ | 0,45 |
| $p_e$ | 0,54 |
| $\kappa$ | 0,29 |
